# Gender differences in adiponectin levels and body composition in older adults: Hallym aging study

**DOI:** 10.1186/1471-2318-14-8

**Published:** 2014-01-25

**Authors:** Hong Ji Song, Sohee Oh, Shanai Quan, Ohk-Hyun Ryu, Jin-Young Jeong, Kyung-Soon Hong, Dong-Hyun Kim

**Affiliations:** 1 Department of Family Medicine, Hallym University Sacred Heart Hospital, College of Medicine, Halym University, Anyang, Korea; 2 Department of Biostatistics, Seoul Metropolitan Government Seoul National University Boramae Medical Center, Seoul, Korea; 3 Department of Social and Preventive Medicine, Hallym University College of Medicine, Chuncheon, Korea; 4 Hallym Research Institute of Clinical Epidemiology, Chuncheon, Korea; 5 Department of Endocrinology, Chuncheon Sacred Heart Hospital, College of Medicine, Hallym University, Chuncheon, Korea; 6 Korea Health Promotion Foundation, Seoul, Korea; 7 Department of Cardiology, Chuncheon Sacred Heart Hospital, College of Medicine, Hallym University, Chuncheon, Korea

**Keywords:** Adiponectin, Older adults, Body composition, Gender difference, Ageing

## Abstract

**Background:**

Body composition changes with ageing can influence the adiponectin concentration. However, the component of body composition that is associated with adiponectin concentrations in older adults remains unclear.

**Methods:**

There were 152 males and 168 females aged 65 years or older that participated in the 2010 Hallym Aging Study (HAS). Body composition (assessed by dual energy X-ray absorptiometry; DXA), anthropometric parameters and adiponectin were obtained from all participants. Multivariate linear regression models assessed the association of body fat percentage, regional muscle and bone mineral contents of body composition and waist/height ratio with adiponectin concentration. Age, albumin, testosterone concentration and metabolic parameters were considered as confounding factors.

**Results:**

In correlation analysis, age was positively associated with adiponectin in males (P < 0.01), but not in females. Fasting glucose, albumin, arm skeletal muscle mass and bone mineral content were negatively associated with adiponectin in males (P < 0.05). Testosterone and leg bone mineral content were negatively associated with adiponectin in females (P < 0.05). In multivariate linear regression models, body fat percentage and albumin (P < 0.05) were negatively associated with adiponectin, and high-density lipoprotein cholesterol (HDL-C) (P < 0.001) and age (P < 0.01) were positively associated with adiponectin in older males. In older females, the only factors that correlated significantly with adiponectin concentration were the homeostasis model assessment of insulin resistance (P < 0.001) and HDL-C (P < 0.05). The waist/height ratio and bone mineral content were not associated with adiponectin in either gender.

**Conclusion:**

Plasma adiponectin levels correlated negatively with body fat percentage in older males but not in older females. The differential results between older males and females suggest that certain gender-specific mechanisms may affect the association between adiponectin and age-related body composition changes.

## Background

Adiponectin, the circulating peptide hormone secreted by adipocytes, reportedly has an important role in insulin resistance, glucose and lipid metabolism and cardiovascular morbidity and mortality [1-4]. In previous studies, adiponectin showed an inverse relationship to adipose tissue mass, especially central adiposity [5-7]. Despite evidence of adiponectin as a cardiovascular risk marker supported by early reports of a strong inverse association with incident coronary heart disease (CHD) in healthy middle-aged males [8], the inverse relationship between adiponectin and CHD was comparatively moderate in other populations [9]. Furthermore, in older populations, higher adiponectin concentrations were associated with greater risk of CHD, stroke or mortality or, conversely, no association was observed between adiponectin and risk of stroke [10-12]. Possible explanations for these conflicting associations were reported as reverse causation from reactive increases [13] or different proportions of high-molecular-weight (HMW) adiponectin [12]. However, the reason for this paradoxical finding in older adults remains unclear.

Visceral adiposity is strongly related to insulin resistance and cardiometabolic disease risk [14,15]. Some researchers reported that not only visceral adipose tissue but also abdominal subcutaneous adipose tissues are both associated with adverse cardiometabolic risk [16,17]. In recent years, research has focused on muscle’s protective effect from metabolic syndrome because higher muscle mass has been reported to be associated with better insulin sensitivity and lower risk of insulin resistance [18,19]. Body composition changes with age [20]. The gender-based difference of body composition change in older adults can influence the adiponectin level.

In this study we determined which body composition component was associated with adiponectin concentration and the gender difference of the association in older adults.

## Methods

### Study population

The Hallym Aging Study (HAS) is a prospective cohort of 1,520 individuals (30% aged 45–64 years and 70% aged 65 years or older) in Chuncheon, a small city in South Korea. Details of the HAS have been published elsewhere [21-24]. The first wave began in 2003 and an in-depth clinical study was started in 2004. The city was divided into 1,408 areas based on the 2000 census and 200 areas were randomly selected. The first-wave participants were selected by systematic sampling: 30% of subjects were sampled from individuals aged 45 to 64 years and 70% were sampled from individuals aged 65 years or older. The first-wave survey in 2003 included 1,520 participants. Among them, 918 had participated in an in-depth clinical study in 2004. Among the 918 participants, 547 agreed to participate in the 2007 follow-up examinations and 382 among the 547 agreed to participate in the 2010 follow-up examinations. We used the 2010 HAS data. A total of 320 participants (152 males and 168 females) aged 65 years or older with anthropometric measurements and body composition analysis constituted the final study population. The Hallym University’s institutional review board approved all protocols and procedures, and informed consent was obtained from all study subjects.

### Data collection, questionnaires and measurements

This investigation was composed of questionnaires, anthropometric measurements and laboratory tests. Questionnaires were administered face-to-face by trained interviewers. Anthropometric measurements and laboratory tests were performed by a clinical team from Chuncheon Sacred Heart Hospital. Age, smoking status, alcohol drinking, regular exercise and past/current medical history were obtained from structured questionnaires. Anthropometric measurements were performed with subjects wearing light clothing and no shoes. Quality control for all measurements was monitored regularly. The height was measured to the nearest 0.1 cm and weight to the nearest 0.1 kg in the upright position. The BMI was calculated as the body weight divided by the height squared (kg/m^2^). Waist circumference was measured at the end of each subject’s normal expiration to the nearest 0.1 cm at the midpoint between the lower end of the 12th rib and the upper end of the iliac crest using anthropometric tape. The waist-to-height ratio was calculated as waist circumference (cm)/height (cm). The body composition including body fat percentage, skeletal muscle mass and bone mineral content was quantified with dual energy X-ray absorptiometry (DXA; Lunar, GE, Fairfield, CT, USA). The systolic blood pressure and diastolic blood pressure were measured in the seated, rested subjects, using a standard protocol. Blood samples were drawn from the antecubital vein in the morning after an overnight fast. Plasma glucose, total cholesterol (T-Chol), triglyceride (TG) levels and high-density lipoprotein cholesterol (HDL-C) were measured using an auto-analyser (Hitachi 747, Hitachi, Tokyo, Japan). Low-density lipoprotein cholesterol (LDL-C) was calculated by the Friedwald equation (LDL-C = T-Chol–(HDL-C + TG/5)). The homeostasis model assessment of the insulin resistance (HOMA-IR) was calculated as follows: fasting serum insulin (μU/mL) × fasting plasma glucose (mg/dL)/405. Glomerular filtration rate (GFR) was estimated based on the 4-variable Modification of Diet in Renal Disease (MDRD) Study equation as follows: eGFR (mL/min/per 1.73 m^2^) = 186 × (serum creatinine (mg/dL)^-1.154^) × (age^-0.203^) × (0.742 if female) × (1.210 if black) [25,26]. Adiponectin measurements were performed from plasma samples stored at -70°C and measured by immunoassay (VersaMax, Molecular Devices, Sunnyvale, CA, USA).

### Statistical analyses

Data are expressed as mean ± standard deviation (SD). The assumption of normality of the data was tested by the Shapiro-Wilk test, and a *p-*value greater than 0.05 indicated the observed distribution of a variable was not statistically different from the normal distribution. Adiponectin, insulin, HDL-C, TG and HOMA-IR values were log transformed, since they were not normally distributed. Comparison of demographic and metabolic variables was performed using Student’s *t*-test. To examine the gender-specific relative contribution of age and body composition, subgroup analyses of males and females were performed using a linear regression model. Relationships between dependent and independent variables were analysed using the Pearson’s correlation test and simple linear regression. Next, the multiple linear regression analysis was used to determine whether the association between the dependent and independent variables of interest remained significant after adjusting for other potentially confounding independent variables.

A *p*-value less than 0.05 was considered statistically significant. All statistical analyses were performed by IBM SPSS Statistics version 20 (IBM Corp., Armonk, NY, USA) and R version 2.15.2 (http://www.r-project.org).

## Results

The subjects showed differences in several metabolic parameters, body composition, comorbidities and health behaviour between males and females (Table 1). Plasma adiponectin, fasting insulin and HOMA-IR were significantly higher in females than in males. BMI, waist/height ratio and body fat percentage were also significantly higher in females than in males. Conversely, total lean body mass, skeletal muscle mass, bone mineral content of limbs and testosterone were significantly higher in males than in females. Regarding health behaviour, males showed a much higher prevalence of smoking and alcohol drinking than females and were more physically active than females.

**Table 1:** General characteristics of the subjects

	**Males (n = 152)**	**Females (n = 168)**	***p*-value**
Age (year)	75.67 ± 5.36	74.44 ± 4.73	0.030
BMI (kg/m^2^)	24.07 ± 2.99	25.26 ± 3.57	0.001
Waist/height ratio	0.54 ± 0.05	0.58 ± 0.07	< 0.001
Body fat (%)	24.89 ± 7.74	34.02 ± 8.52	< 0.001
Fat mass (g)	16935.33 ± 4953.03	19463.03 ± 5665.38	< 0.001
SBP (mmHg)	137.8 ± 16.12	138.95 ± 16.74	0.535
DBP (mmHg)	78.04 ± 9.05	77.77 ± 8.44	0.781
Fasting glucose (mg/dL)	100.68 ± 25.76	98.82 ± 29.44	0.550
HbA_1_C (%)	5.96 ± 0.91	5.93 ± 0.75	0.798
Fasting insulin (μU/mL)	4.48 ± 2.25	5.87 ± 2.01	0.002
HOMA-IR	1.09 ± 2.46	1.40 ± 2.16	0.009
LDL cholesterol (mg/dL)	111.32 ± 34.62	115.69 ± 32.98	0.251
HDL cholesterol (mg/dL)	46.99 ± 1.30	47.94 ± 1.28	0.411
Triglyceride (mg/dL)	120.30 ± 1.75	127.74 ± 1.70	0.320
Albumin (g/dL)	4.47 ± 0.36	4.47 ± 0.31	0.923
hs-CRP (mg/L)	0.38 ± 1.4	0.22 ± 0.65	0.194
Testosterone (ng/ml)	5.20 ± 1.86	0.12 ± 0.13	< 0.001
e-GFR (mL/min/per 1.73 m^2^)	78.54 ± 18.14	85.42 ± 19.18	0.001
Adiponectin (μg/mL)	8.00 ± 1.75	10.38 ± 1.67	< 0.001
Body composition			
Total LBM (gram)	43924.49 ± 6008.97	34271.89 ± 4551.19	< 0.001
Arm LBM (gram)	4962.42 ± 880.34	3614.25 ± 747.13	< 0.001
Leg LBM (gram)	13828.23 ± 2326.93	10294.68 ± 1699.85	< 0.001
Arm SM (gram)	4592.98 ± 856.51	3382.68 ± 726.57	< 0.001
Leg SM (gram)	12933.74 ± 2192.03	9681.23 ± 1591.48	< 0.001
Arm BMC (gram)	344.3 ± 72.24	221 ± 82.16	< 0.001
Leg BMC (gram)	894.49 ± 168.19	613.45 ± 143.95	< 0.001
Comorbidities on treatment			
Hypertension (%)	75 (49.34)	90 (53.57)	0.450
DM (%)	26 (17.11)	22 (13.1)	0.316
Dyslipidemia (%)	63 (42.28)	107 (63.69)	< 0.001
CVA (%)	15 (9.87)	11 (6.55)	0.278
MI (%)	14 (9.21)	5 (2.98)	0.018
Lifestyle			
Smoking (pack/year)	30.27 ± 28.56	0.65 ± 4.23	< 0.001
Alcohol (g/week)	152.74 ± 362.32	7.94 ± 33.44	< 0.001
Exercise (%)			< 0.001
< 3 times/week	105 (69.08)	152 (90.48)	
3-4 times/week	14 (9.21)	7 (4.17)	
> 4 times/week <	33 (21.71)	9 (5.36)	

*Abbreviations*: *BMI* body mass index, *SBP* systolic blood pressure, *DBP* diastolic blood pressure, *HOMA-IR* homeostasis model assessment of insulin resistance, *LDL* low-density lipoprotein, *HDL* high-density lipoprotein, *hs-CRP* high-sensitivity C-reactive protein, *e-GFR* estimated glomerular filtration rate, *LBM* lean body mass *SM* skeletal muscle mass, *BMC* bone mineral content, *DM* diabetes mellitus, *CVA* cerebrovascular accident, *MI* myocardial infarction. All values are means ± SDs or n (%). *p*-values were calculated by Student’s *t*-test or Chi-square test. Adiponectin, insulin, HDL, TG and HOMA-IR were log transformed because they were not normally distributed. The data describing comorbidities and treatments were obtained from structured questionnaires.

In correlation analysis, the most significant correlations were observed between adiponectin and HDL-C, triglyceride and HOMA-IR in both genders. Gender differences in older adults were observed in the relationship between adiponectin concentrations and phenotypes, including metabolic parameters and body composition (Table 2). Age was positively associated with adiponectin in males (P < 0.01), but there was no significant relationship between adiponectin and age in females. Fasting glucose and albumin were negatively associated with adiponectin in males (P < 0.05), but not in females. Testosterone was negatively associated with adiponectin in females (P < 0.05), but not in males. Arm skeletal muscle mass and bone mineral content were negatively associated with adiponectin in males (P < 0.05), but not in females. Leg bone mineral content was negatively associated with adiponectin in females (P < 0.05), but not in males in univariate analysis.

**Table 2:** Relationships between adiponectin concentrations and phenotypes including metabolic parameters and body composition

	**Males**		**Females**	
	Correlation coefficient (95% CI)*	***p*-value**	Correlation coefficient (95% CI)*	***p*-value**
Age (year)	0.250 (0.093, 0.395)	0.002	0.129 (−0.023, 0.275)	0.095
BMI (kg/m^2^)	−0.305 (−0.444, −0.152)	< 0.001	−0.294 (−0.427, −0.150)	< 0.001
Waist/height ratio	−0.202 (−0.353, −0.042)	0.014	−0.304 (−0.435, −0.160)	< 0.001
Body fat (%)	-0.295 (-0.435, −0.140)	< 0.001	−0.295 (−0.428, −0.151)	< 0.001
Fat mass (g)	−0.311 (−0.450, −0.157)	<0.001	−0.329 (−0.458, −0.185)	< 0.001
SBP (mmHg)	0.101 (−0.061, 0.257)	0.222	−0.059 (−0.208, 0.093)	0.449
DBP (mmHg)	0.067 (−0.095, 0.225)	0.419	−0.045 (−0.195, 0.107)	0.559
Fasting glucose (mg/dL)	−0.174 (−0.326, −0.014)	0.033	−0.095 (−0.243, 0.057)	0.220
HbA_1_C (%)	−0.205 (−0.354, −0.045)	0.012	−0.167 (−0.310, −0.016)	0.031
Fasting insulin (μU/mL)	−0.329 (−0.465, −0.178)	< 0.001	−0.428 (−0.544, −0.295)	< 0.001
HOMA-IR	−0.339 (−0.474, −0.188)	< 0.001	−0.425 (−0.541, −0.292)	< 0.001
LDL cholesterol (mg/dL)	−0.096 (−0.253, 0.066)	0.244	−0.033 (−0.183, 0.119)	0.673
HDL cholesterol (mg/dL)	0.425 (0.284, 0.548)	< 0.001	0.354 (0.214, 0.480)	< 0.001
Triglyceride (mg/dL)	−0.399 (−0.526, −0.255)	< 0.001	−0.362 (−0.487, −0.223)	< 0.001
Albumin (g/dL)	−0.184 (−0.335, −0.024)	0.025	−0.087 (−0.236, 0.065)	0.260
hs-CRP (mg/L)	−0.010 (−0.170, 0.151)	0.905	0.009 (−0.143, 0.160)	0.909
Testosterone (ng/ml)	0.122 (−0.040, 0.277)	0.139	−0.183 (−0.325, −0.032)	0.018
e-GFR (mL/min/per 1.73 m^2)^	−0.016 (−0.177, 0.145)	0.842	−0.09 (−0.238, 0.062)	0.247
Body composition				
Total LBM	−0.123 (−0.278, 0.039)	0.136	−0.134 (−0.279, 0.018)	0.084
Arm LBM	−0.200 (−0.350, −0.041)	0.014	-0.163 (−0.307, −0.012)	0.034
Leg LBM	−0.117 (−0.273, 0.045)	0.156	−0.136 (−0.282, 0.015)	0.078
Arm SM	−0.229 (−0.376, −0.071)	0.005	−0.146 (−0.291, 0.006)	0.059
Leg SM	−0.113 (−0.269, 0.049)	0.170	−0.124 (−0.270, 0.028)	0.110
Arm BMC	−0.187 (−0.338, −0.027)	0.023	−0.107 (−0.255, 0.046)	0.169
Leg BMC	−0.145 (−0.299, 0.016)	0.077	−0.24 (−0.378, −0.092)	0.002
Comorbidities on treatment				
Hypertension		0.652		0.013
Yes	2.098 (1.966, 2.230)		2.249 (2.148, 2.351)	
No	2.057 (1.938, 2.176)		2.442 (2.330, 2.555)	
DM		0.002		0.005
Yes	1.770 (1.600, 1.940)		2.056 (1.851, 2.261)	
No	2.142 (2.045, 2.239)		2.382 (2.301, 2.462)	
Dyslipidemia		< 0.001		0.002
Yes	1.815 (1.691, 1.939)		2.248 (2.157, 2.338)	
No	2.269 (2.160, 2.379)		2.499 (2.368, 2.630)	
CVA		0.188		0.145
Yes	1.898 (1.705, 2.090)		2.123 (1.808, 2.438)	
No	2.097 (2.002, 2.193)		2.354 (2.275, 2.433)	
MI		0.630		0.104
Yes	2.006 (1.656, 2.356)		1.975 (1.497, 2.453)	
No	2.084 (1.993, 2.175)		2.350 (2.273, 2.428)	
Lifestyle				
Smoking (pack/year)	0.102 (−0.060, 0.259)	0.215	0.134 (−0.018, 0.279)	0.084
Alcohol (g/week)	−0.009 (−0.170, 0.152)	0.912	0.006 (−0.145, 0.157)	0.938
Exercise		0.716		0.451
< 3 times/week	2.100 (1.991, 2.208)		2.324 (2.243, 2.405)	
3-4 times/week	1.982 (1.765, 2.198)		2.548 (2.204, 2.891)	
> 4 times/week	2.047 (1.849, 2.245)		2.430 (2.125, 2.734)	

*Mean (95% CI) for categorical variables. *Abbreviations*: *BMI* body mass index, *SBP* systolic blood pressure, *DBP* diastolic blood pressure, *HOMA-IR* homeostasis model assessment of insulin resistance, *LDL* low-density lipoprotein, *HDL* high-density lipoprotein, *hs-CRP* high-sensitivity C-reactive protein, *e-GFR* estimated glomerular filtration rate, *LBM* lean body mass, *SM* skeletal muscle mass, *BMC* bone mineral content, *DM* diabetes mellitus, *CVA* cerebrovascular accident, *MI* myocardial infarction, *CI* confidence interval. Adiponectin, insulin, *HDL* TG and HOMA-IR were log transformed because they were not normally distributed. The data describing comorbidities and treatments were obtained from structured questionnaires.

Multiple linear regression models assessed the association of body fat percentage, regional muscle mass and bone mineral content of body composition and waist/height ratio with adiponectin concentration (Table 3). After adjusting the metabolic parameters, body fat percentage and albumin (P < 0.05) were negatively associated with adiponectin in males, but not in females. HDL-C (P < 0.001) and age (P < 0.01) were positively associated with adiponectin in older males. In older females, the only factors that correlated significantly with adiponectin concentration were HOMA-IR (P < 0.001) and HDL-C (P < 0.05). The waist/height ratio and bone mineral content were not associated with adiponectin in either gender.

**Table 3:** Multiple linear regressions of adiponectin (dependent variables) with age and body composition for older males and females

	**Males**				**Females**			
**Parameter**	**Model 1**	**Model 2**	**Model 3**	**Model 4**	**Model 1**	**Model 2**	**Model 3**	**Model 4**
Age	0.026^b^	0.018^a^	0.018^a^	0.024^b^	0.014	0.004	0.001	0.011
Body fat (%)	-	−0.021^b^	−0.020^b^	−0.013^a^	-	−0.011^a^	−0.011^a^	−0.003
Arm SM (g)	-	0.000	0.000	0.000	-	0.000	0.000	0.000
Arm BMC (g)	-	−0.002	−0.001	−0.001	-	0.000	0.000	0.000
Leg SM (g)	-	0.000	0.000	0.000	-	0.000	0.000	0.000
Leg BMC (g)	-	0.000	0.000	0.000	-	−0.001^a^	−0.001^a^	−0.001
Waist/Height	-	−0.712	−0.496	0.955	-	−0.565^a^	−0.424^a^	−0.516
Albumin	-	-	−0.200	−0.251^a^	-	-	−0.041	−0.009
Testosterone	-	-	0.027	0.028	-	-	−0.630	−0.412
HOMA-IR	-	-	-	0.008	-	-	-	−0.172^b^
HDL-Cholesterol	-	-	-	0.876^c^	-	-	-	0.423^a^
Triglycerides	-	-	-	−0.127	-	-	-	−0.153
Model adjusted R^2^	0.056	0.152	0.167	0.322	0.011	0.136	0.142	0.285

*Abbreviations*: *SM* skeletal muscle mass, *BMC* bone mineral content, *HDL* high-density lipoprotein, *HOMA-IR* homeostasis model assessment of insulin resistance. Values are standardized ß-coefficients. Model 1 includes age. Model 2 includes Model 1 plus body composition parameters and waist/height ratio. Model 3 includes the Model 2 variables plus albumin and testosterone. Model 4 includes the Model 3 variables plus metabolic variables. ^a^0.05 > P ≥ 0.01. ^b^0.01 > P ≥ 0.001. ^c^P < 0.001. “−” indicates not included in model. Adiponectin, HOMA-IR, HDL and triglycerides were log-transformed for analyses.

## Discussion

In the present analysis, a gender difference was found in the relationship between adiponectin concentration and body fat in older adults. Age and gender are two of the most important confounding or effect modifying factors for most diseases. In particular, gender-specific body adiposity and age-related changes in body composition can influence metabolic profiles and cardiovascular diseases [20]. Increased abdominal fat is associated with insulin resistance and atherogenic metabolic profiles [27]. Adiponectin has been reported to have an important role in glucose and lipid metabolism [1-3] and the most pronounced correlations were observed between adiponectin and HDL-C, triglycerides and HOMA-IR in the present study. The inverse relationship of circulating adiponectin to adipose tissue mass, especially central adiposity is well known [5-7]. However, paradoxical results have been reported in older populations, such as higher adiponectin concentration associated with greater risk of CHD, stroke or mortality [10-12]. In the result of multiple linear regression analysis for the relationship between adiponectin concentrations and body composition in our study, the body fat percentage was negatively associated with adiponectin in males, but not in females (Table 3). Except for a small contribution of body fat percentage in males, the waist/height ratio and body composition, including muscle and bone mineral content, had no relationship with adiponectin levels in either gender. In the previous study, bone mineral density was negatively associated with adiponectin levels in males > 60 years with a BMI > 27 kg/m^2^[28]. However, in that study, the influence of waist circumference and body composition, including body fat and muscle, was not adjusted.

In the previous studies, age was positively associated with adiponectin [12,29,30]; however, in our analysis of older adults, age was positively associated with adiponectin in males, but not in females (Tables 2 and 3). Adipocytes and their products, including leptin and adiponectin, play an important role in the interaction between energy balance and the reproductive axis [31]. In the previous studies, the association of adiponectin with testosterone and estradiol was inconsistent. In a Caucasian population, higher levels of testosterone and lower estradiol concentrations predicted higher adiponectin levels in both genders [32]. However, in an Asian population, adiponectin levels were negatively correlated with testosterone but were not correlated with estradiol in postmenopausal females (more than 1 year since menopause) [33]. In our study, testosterone and adiponectin showed a significant negative association in the correlation analysis for older females. However, in multiple linear regression analysis, the correlation between testosterone and adiponectin in females was insignificant, even though the estimate of correlation changed from −0.183 to −0.412. The results of our study suggested that hormonal changes according to age and gender were not important factors that influenced the paradoxical finding of adiponectin in older Asian adults. In an *in vitro* study using human fat cells, increasing concentrations of testosterone or estradiol did not influence adiponectin mRNA expression and secretion or the intracellular levels of high-, middle-, and low-molecular-weight adiponectin multimers. However, stimulation with human male and female serum downregulated adiponectin expression, with male serum exerting significantly stronger inhibitory properties than female serum, suggesting the presence of a serum factor that causes the gender dimorphism in circulating adiponectin levels [34].

One strength of our study was that the data of older males and females were analysed separately. In several other studies, researchers did not analyse the male and female data separately, considering gender difference as a confounding factor [12,35], or they analysed data from males only [10,11]. However, our results suggested that gender difference was not only a confounding factor, but also an effect modifier in older adults; therefore, data from older males and females should be analysed separately. Another strength of our study was that we considered not only weight but also body composition using DXA and waist circumference in older adults. In previous studies, adiponectin was associated with weight change only in females [36] or was not associated with changes in weight in older adults [37]. However, in those studies, the amount of fat was not examined, which may explain the inconsistency between our results and previous studies.

The weakness of our study was the relatively small sample size. To minimise the effects of this limitation, participants were selected by systematic sampling from 200 areas that were selected randomly in Chuncheon, a small city in South Korea. Nevertheless, the sampling method cannot completely overcome the limitation from small sample size. Compared with other populations, the contribution of body fat and muscle to the adiponectin level in an older population was very small or insignificant after multiple regression analysis. Therefore, a larger study population is needed to determine the small but significant contribution of body fat and muscle to adiponectin levels in an older population.

## Conclusion

Plasma adiponectin levels correlated negatively with body fat percentage in older males but not in older females. The associations of adiponectin with some other factors also differed between older males and females. This suggests that certain sex-specific mechanisms may affect the association between adiponectin and age-related changes in body composition.

## Competing interests

The authors declare that they have no competing interests.

## Authors’ contributions

HJS suggested the study. HJS, SO and DHK designed the study and developed the study protocol. SO and HJS analysed the data. All authors interpreted the results. HJS drafted the manuscript. All authors contributed to the critical revision of the manuscript. DHK has full access to all of the data in the study and takes responsibility for the integrity of the data and the accuracy of the data analysis. All authors read and approved the final manuscript.

## Pre-publication history

The pre-publication history for this paper can be accessed here:

http://www.biomedcentral.com/1471-2318/14/8/prepub
